# Predictors of Developing Hepatocellular Carcinoma in Treated HCV-Carriers in Morocco according to University Hospital Experience

**DOI:** 10.1155/2013/438306

**Published:** 2012-12-23

**Authors:** Younès Cherradi, Rajaa Afifi, Hanaa Benbrahim, Wafaa Essamri, Imane Benelbarhdadi, Fatima Zahra Ajana, Hadj Omar El Malki, Mustapha Benazzouz, Abdellah Essaid

**Affiliations:** ^1^Department of Hepatogastroenterology (Medicine C), Ibn Sina Hospital, Rabat, Morocco; ^2^Biostatical, Clinical Research and Epidemiological Laboratory (LBRCE), Medical School, University Mohammed Vth Souissi, Rabat, Morocco

## Abstract

*Introduction*. Hepatitis C is the first major cause for HCC in Morocco. Antiviral treatment reduces the risk of developing HCC but few cases of HCC in HCV-treated patients were reported. We aimed to define this population's features and to identify predictive factors of developing HCC. *Patients and Methods*. We included all HCV carriers who developed HCC after antiviral treatment from January 2002 to April 2010. We compare HCV-treated patients with no developed HCC to HCC population using khi-2 and Fisher Exact analysis. *Results*. 369 HVC-treated patients were considered, and 20 HCC were reported. The risk of HCC was not significant according to gender and genotypes (resp., *P* = 0.63 and *P* = 0.87). Advanced age and severe fibrosis were significant risk factors (resp., *P* = 0.003 and *P* = 0.0001). HCC was reported in 2.6% of sustained virological responders versus 12.5% of nonresponders (*P* = 0.004). *Conclusion*. In our series, 5% of previously treated patients developed an HCC. Advanced age and severe fibrosis at HCV diagnosis are predictive factors of HCC occurrence. Sustained virological response reduces considerably the risk of HCC occurrence but screening is indicated even after SVR.

## 1. Introduction

Hepatocellular carcinoma (HCC) is one of the most common malignant tumors in the world. It is the main type of primary liver cancers and the third most common cause of cancer mortality worldwide [1]. The epidemiology of HCC is variable according to the geographic area because of differences in the repartition of major causative factors. In countries where hepatitis C virus infection is endemic such as Japan and Egypt, high prevalence of HCV infection is reported among people with HCC. In Morocco, according to our department register and the results of a regional study [2], the major risk factor for hepatocellular carcinoma is chronic hepatitis especially hepatitis C virus infection. Hepatocellular carcinoma is closely associated to liver cirrhosis, which is a true precancerous state. Hepatitis viruses B and C contribute to this condition if not treated or diagnosed late [3]. Hepatocarcinogenesis is a long and heterogeneous process and there is still much to understand. The combination of pegylated interferon to ribavirin given for 24 or 48 weeks—according to genotype—has been retained for long years ago as the only consensual and standard treatment of chronic hepatitis C before the development of antiproteasis and antipolymerases for selected patients. It is established too that antiviral treatment limits fibrosis progression and reduces risk of developing HCC but few cases of  HCC in HCV carriers are still reported even after antiviral treatment. The aim of this study is to define this population's features and to identify predictive factors of developing HCC. The major endpoint is to define patients who must be screened carefully after antiviral therapy in order to early diagnosis and more prognosis involvement.

## 2. Patients and Methods

It is a monocentric, retroprospective, and analytic study. It concerns our entire patients department with hepatitis C virus infection who developed HCC after antiviral treatment. All participants were treated by pegylated interferon associated to ribavirin. They were all tested for HCC prior to antiviral therapy. At study onset, included patients had no HCC and they were not coinfected by hepatitis B. Diagnosis of HCC was based on histological confirmation when biopsy was performed or on morphological noninvasive criteria (Barcelona criteria) for cirrhotic patients where biopsy was not necessary. Considered parameters were age of HCV diagnosis, gender, baseline viral loads (BVL), genotype, necro-inflammatory activity, and fibrosis degrees at the beginning of antiviral treatment. Fibrosis and histological activity were estimated according to METAVIR score (*F* > 2 was considered as an advanced stage of fibrosis). We compare initially HCV-treated patients who did not develop HCC to patients who developed HCC using khi-2 and Fisher-Exact analysis. In a second step, we were particularly interested to special cases of sustained virological responders who developed HCC. The pretherapeutic parameters previously detailed were collected and analyzed too. We compare sustained virological responders who did not develop HCC to the others using Pearson Chi-square test. Follow-up time was at least 03 years.

## 3. Results

Three hundred sixty-nine HCV-treated patients were considered from January 2002 to April 2010. *Twenty* HCC were reported with 12 females (60%) and 8 males (40%), ranging in age between 40 and 72 years; the mean age is 61 years. The mean time of HCC occurrence is 5 ± 2 years. 53% of patients were HCV-1, 47% were HCV-2, and 94, 6% had severe fibrosis at the beginning of treatment (Table 1). Considered all our treated patients, sustained virological response (SVR) was achieved in 70%. The comparison of HCC occurrence in non-SVR patients and sustained virological responders shows significant results: HCC was reported in 2.6% of sustained virological responders versus 12.5% of non-SVR patients (*P* = 0.004) which demonstrate that SVR reduces significantly the risk of developing HCC. The risk of HCC was not significant according to gender and genotypes (resp., *P* = 0.63 and *P* = 0.87). Advanced age and severe fibrosis were significant risk factors (resp., *P* = 0.003 and *P* = 0. 0001) (Table 2).

Of the 20 patients who developed HCC, six were previously sustained virological responders. They were 3 males and 3 females, ranging in age between 51 and 70 years. Mean age is 60 years. *Five* patients were HCV-2 and one patient was HCV-1. *All* patients were in severe fibrosis (*F*3-*F*4). Time of follow-up ranges between 3 and 9 years with a median of 6 years. The risk of HCC occurrence in sustained virological responders seems not to be significant according to gender, genotypes, and initial high viral loads (resp., *P* = 1, *P* = 0.4 and *P* = 0.57). High necroinflammatory activity did not influence hepatocarcinogenesis. Severe fibrosis was the only significant risk factor (*P* = 0.01) in our real life experience (Table 3).

## 4. Comment

Hepatitis C is a real public health trough the world. There are more than 170 million people infected worldwide [4]. Approximately, 80% of HCV carriers develop chronic hepatitis C [4]. About 20% of these patients will develop severe chronic hepatitis C and cirrhosis which is considered as a precancerous condition predisposing to development of hepatocellularcarcinoma.

Treatment of hepatitis C virus infection has been developed rapidly and, in twenty years, SVR rates increased from 6% to 60% [5]. Actually, and in expectation of protease and polymerase inhibitors for particular situations, the association of pegylated interferon and ribavirin is retained as standard of care in HCV-chronically-infected patients. Effectiveness of antiviral treatment is proved and in our department patients, where genotypes 1 and 2 are more prevalent, SVR is achieved in 70% of all patients which is very interesting result. Actually, there is strong evidence that eradicate HCV infection improves the prognosis of patients and limits fibrosis progression which reduces—but does not abolish—the risk of developing hepatocellular carcinoma [6]. It means that possibility of HCC development is still present in treated patients and screening is still indicated even after SVR especially when HCV was diagnosed late (>50 years old in our study) with severe fibrosis (*F*3-*F*4). In another sense, SVR is certainly a significant protector factor. It was demonstrated that in patients with chronic hepatitis B or C, antiviral treatment in a noncirrhotic stage is protective for HCC occurrence in responders, probably by prevention of cirrhosis development [7]. When cirrhosis is already present, the protective effect is less clear. For cirrhosis due to hepatitis C, some studies—especially in Japan—showed a protective role of interferon-alfa [7, 8]. Virological response, but also merely biochemical response, seems to be associated with a lower risk of development of HCC [7], but screening is indicated even after SVR. In our study, predictors of HCC occurrence in all treated HCV carriers are advanced age (>50 years old), severe fibrosis, and non-SVR; the protective role of SVR was established. In the special case of sustained virological responders, severe fibrosis is the only significant predictor of developing HCV. we conclude that patients with advanced fibrosis must be considered carefully with necessity of continuous screening even after SVR.

## 5. Conclusion

Considering our real life experience, significant predictors of HCC occurrence in treated HCV carriers are advanced age, severe fibrosis, and non-SVR. In other side, this study confirms the fact that SVR reduces significantly the risk of HCC development; so, screening is indicated even after SVR especially in patients with severe fibrosis.

## Figures and Tables

**Table 1:** General features of HCV-treated patients with developed HCC.

Gender	
Male	08
Female	12
Mean age	61 years old.
Mean time of HCC occurrence	5 ± 2 years
HCV genotypes	
HCV-1	53%
HCV-2	47%
Fibrosis (METAVIR score)	
Severe	94.6%
Reduced	5.4%
Response to antiviral treatment	
Sustained virological responders	06
Non-SVR patients	14

**Table 2:** Predictors of developing HCC in treated HVC carriers.

	*P*
Gender	*P* = 0.63
Age of diagnosis > 60 y.o	*P* = 0.003
Genotype	*P* = 0.87
Initial fibrosis	*P* = 0.0001
Non-SVR	*P* = 0.004

**Table 3:** Fibrosis is significantly associated to HCC occurrence in sustained virological responders ∗∗∗∗.

	Patients with developed HCC		Patients with no HCC	*P*
Gender				
Male	3.3%	Versus	96.7%	*P* = 1
Female	3.4%	Versus	96.6%	
Age of diagnosis				
Age ≤ 55	1%	Versus	99%	*P* = 0.1
Age > 55	5.7%	Versus	94.3%	
HCV Genotypes				
1	1.3%	Versus	98.7%	*P* = 0.4
2	4.7%	Versus	95.3%	
Baseline viral loads (BVL)				
High BVL	3%	Versus	97%	*P* = 0.57
Low BVL	1.1%	Versus	98.9%	
Necroinflammatory activity				
*A* ≤ 2	3.2%	Versus	96.8%	*P* = 0.4
*A* > 2	6.3%	Versus	93.7%	
Fibrosis∗∗∗∗				
*F* ≤ 2	0%	Versus	100%	*P* = 0.01
*F* > 2	8.8%	Versus	91.2%	
